# Supplementary material for: Are carbon emissions trading and green financial instruments synergistic? -Comprehensive quantitative research based on content analysis
Source: PLoS One. 2024 Mar 7;19(3):e0298601. doi: 10.1371/journal.pone.0298601 (PMC10919601; doi:10.1371/journal.pone.0298601)
Supplement: S1 Data — (ZIP) [file pone.0298601.s001.zip › Data/地级市Z_F工作报告.zip!/║╙─╧/╫ñ┬φ╡Ω╩╨/└┤╫╘╣1⁄2╓┌║┼í╛┬φ┐╦╩2╛▌═°í┐_2014.docx]

各位代表：

现在，我代表市人民政府，向大会报告工作，请予审议，并请各位政协委员和其他列席人士提出意见。

一、2013年工作回顾

过年的一年，面对复杂的经济形势和繁重的发展任务，在省委、省政府和市委的正确领导下，全市上下深入贯彻党的十八大和十八届三中全会精神，认真落实国家、省一系列政策措施，把稳定经济运行、促进经济回升作为首要任务，坚持大招商、一举求多效，加快城镇化、一发动全身，打造新优势、一优带百通，叫响“三个一”、谋求新作为，较好地完成了市三届人大三次会议确定的目标任务。

(一)经济运行总体平稳。年初由于受国内外宏观经济的影响，我市经济下行压力明显加大，特别是工业运行出现了近年来少有的困难局面。面对这一严峻形势，我们坚持把稳增长摆在突出位置，强力推进项目建设，深入开展大招商活动，强化企业服务，优化发展环境，搞好要素保障，从第三季度起全市经济稳中趋升，全年总体保持平稳增长。初步核算，全市完成生产总值1542亿元，增长9.5%；规模以上工业增加值489.9亿元，增长14.7%；财政总收入113.6亿元，其中地方公共财政预算收入71.9亿元，增长22.1%；社会消费品零售总额585.5亿元，增长13.9%；金融机构存款、贷款余额分别增长21.9%、19.2%；居民消费价格上涨2.7%；城镇居民人均可支配收入19431元，农民人均纯收入7483元，分别增长10%和12.7%；节能减排完成省定目标。

(二)开放改革成效明显。坚持实施开放带动战略，突出主导产业、战略性新兴产业和招大引强，瞄准长三角、珠三角、京津环渤海等重点区域，全年举办16次重大产业招商活动，签约亿元以上项目364个，总投资2138亿元。深入开展迎盛会、大招商活动，中国“农洽会”取得圆满成功，我市共签约亿元以上项目78个，总投资649亿元。全年实际到位市外资金330亿元，增长30%；实际利用外资3.16亿美元，增长16.9%；完成进出口4.04亿美元，增长48.6%，增速位居全省第一，其中出口3.2亿美元，增长47.7%。同时，积极推进各项改革，国有企业脱钩改制扎实推进，市直86户局属企业实现脱钩，46户基本完成改制；行政审批、医药卫生、交通运输、财税、金融、文化等改革深入推进。

(三)项目建设实现突破。深入开展项目建设推进年活动，认真落实项目建设周例会、月通报和领导分包、联审联批等制度，去年共召开41次周例会，解决了一大批制约项目建设的突出问题。全年分四批集中开工229个亿元以上重点项目，总投资1128亿元，年度投资256亿元。全年211个市重点项目完成投资460.3亿元，增长68%。产业集聚区基础设施进一步完善，主导产业逐步显现，全年完成规模以上工业增加值290亿元，占全市规模以上工业增加值的59.2%。全市完成固定资产投资1014.9亿元，增长24.1%，增速居全省第三位，其中产业集聚区固定资产投资713.8亿元，增长29.6%，实现了年初提出的“两个突破”目标。

(四)城市建设快速推进。按照“一中心五组团”战略布局，以中心城区为龙头，统筹推进中心城区、县城、重点镇建设，全市城镇化率达到34.9%，较上年提高1.45个百分点。驻马店城市总体规划已经省政府批准，综合交通、公共服务、绿地系统等八项专项规划和城乡一体化示范区空间战略规划编制完成。募集城投债资金13亿元、利用兴业银行贷款20亿元，引进社会资金参与城市综合开发，七大功能片区、十项城建重点工程顺利推进，全年完成城建基础设施投资61亿元，较上年增长80%。城建重点项目顺利实施，开源大道改造及中原大道中段、金雀路西段、雪松大道西段等建成通车；铜山大道和中原大道北段、前进大道北段、纬十路等开工建设；市第二污水处理厂建成投用，第三污水处理厂加快推进，建筑垃圾处理二期工程投入运营，完成了练江河沿岸14个排污口治理。商务中心区和特色商业区建设稳步推进，全市完成投资85亿元。理顺了中心城区管理体制，形成了“两级政府、三级管理、四级网络”城市管理新格局，城市精细化管理水平进一步提升。开展了中心城区征地拆迁安置集中整治，完成征地1.54万亩，拆迁250万平方米；“双违”整治专项行动有序推进。各县也谋划实施了一批城市综合体和片区开发项目，县城建设全面提速，基础设施不断完善，城镇承载力明显提升。

(五)现代农业稳步发展。全年粮食总产141.4亿斤，增长0.67%，实现了“十连增”，被农业部评为“全国粮食生产先进单位”。大力实施高标准粮田“百千万”工程，新增高标准粮田127万亩，走在了全省前列。大力推进现代畜牧业发展，新建标准化规模养殖场105个，肉蛋奶产量分别增长3.6%、1.5%、0.1%，“夏南牛新品种培育与应用”荣获国家科技进步二等奖。积极推进现代农业产业化集群培育工程，全市农业产业化组织8200多家，农民合作组织达到7314家，市级以上农业产业化龙头企业达到316家。农村生产生活条件持续改善，全年改造建设农村公路336公里，新增农村沼气用户1万户，解决了58万农村居民饮水安全和13.9万贫困人口脱贫问题。稳步推进新农村建设，累计投入资金70多亿元，建成房屋2万多套，搬迁群众4万多人。1月18日汪洋副总理在我市调研时，对我市粮食生产、高标准粮田建设、扶贫开发等工作给予了充分肯定。

(六)民生得到持续改善。进一步加大民生投入，全市各项民生支出237.3亿元，占财政支出的76.9%。社会事业全面发展，新建改扩建幼儿园141所、农村中小学75所；职教园区建设成效明显，完成投资12亿元以上，高级技工学校实现整体搬迁，申办驻马店技师学院和幼儿师范升专通过专家评审，农校、卫校、幼儿师范项目建设加快推进，黄淮学院转型发展走在全国前列。医疗卫生事业“三年九大攻坚计划”全面实施，一批重大疾病控制、县级医院和乡镇卫生院改扩建项目相继完成，人口自然增长率控制在6.5‰以内。科技服务能力不断提高，全年新增省级企业研发中心4家，组织实施各类科技计划项目222项，实施重大科技专项5个，建成昊华骏化博士后工作站，我市被评为全国科技进步先进市。就业和社会保障工作得到加强，完成各类职业技能培训17.6万人次，新增农村劳动力转移就业8.1万人，城镇新增就业9.7万人，失业人员再就业4.1万人；企业退休人员养老金、城乡低保、农村五保对象补助标准得到提高，城乡居民社会养老保险实现全覆盖，新农合和城镇居民医保参保率达到98.8%和93%；开工建设保障性住房2.72万套，基本建成3.1万套。广电基础设施标准不断提高，完成城区有线电视数字化改造10万余户。中心城区六件惠民实事进展顺利，市群艺馆、博物馆、图书馆免费开放，18条道路照明、4个路口改造及冷水河、骏马河景观水系改造工程全部完成，4座过街天桥和3个小游园开工建设，市二中北校区建成投用，十五小、实验幼儿园等8所学校及市残疾人康复中心项目加快推进。107国道顺利撤站回归公益，还路于民。平安驻马店建设持续深化，社会管理机制不断完善，信访形势总体平稳，安全事故起数和死亡人数实现双下降，人民群众安全感进一步提升，社会大局和谐稳定。

一年来，我们自觉接受人大监督、政协民主监督和社会监督，认真办理人大代表建议和政协提案。切实加强民主法制建设，深入推进普法教育和依法治理，广泛开展精神文明创建活动，成功创建“省级文明城市”。切实加强政府自身建设，扎实推进“两转两提”，努力打造法治政府、服务政府、高效政府、创新政府和廉洁政府。高度重视驻驻部队和国防后备力量建设，深入开展拥军优属、拥政爱民和军民共建活动，军政军民团结的良好局面巩固发展。民族宗教、旅游、统计、金融、审计、气象、史志、档案、侨务、人防、红十字、妇女儿童等各项工作都取得了新的成绩，先后获“中国十大最具投资潜力旅游目的地”、“最具投资竞争力金融服务城市”等荣誉称号；在中国城市商业信用环境指数排名中，我市居全国77位、全省第2位。

各位代表!在去年严峻复杂的形势下，取得这些成绩确实不易。这是省委、省政府和市委正确领导的结果，是市人大、政协大力支持的结果，是全市人民奋力拼搏的结果。在此，我代表市政府向工作在各条战线上的工人、农民、知识分子、干部、驻驻部队指战员、武警官兵、公安民警，向所有关心支持政府工作的各位人大代表、政协委员、离退休干部、民主党派、工商联和无党派人士，表示崇高的敬意和衷心的感谢!

各位代表!在肯定成绩的同时，我们也清醒地看到，当前我市经济社会发展还面临很多困难和挑战。一是经济回升向好的基础还不稳固，下行压力依然很大，主要经济指标增速缓慢；部分工业品价格下跌，生产成本上升，融资问题突出，企业经营面临较大挑战；受营改增、结构性减税等政策因素影响，公共财政预算收入增速减缓。二是长期制约我市经济发展的结构性矛盾比较突出，经济总量小，工业化、城镇化水平低，产业结构不合理，一产大而不强，二三产业发展滞后。三是社会事业欠账较多，公共服务水平低，一些涉及群众切身利益的就业、就学、就医、住房等问题尚未得到很好解决，收入分配、环境保护等领域还存在一些群众反映强烈的问题。四是一些政府工作人员的能力素质与新形势、新任务的要求不相适应，作风不实、服务不优、为政不廉等问题仍不同程度存在。对此，我们一定高度重视、认真解决，努力开创政府工作的新局面。

二、2014年政府工作主要任务

今年是全面贯彻落实党的十八届三中全会精神、全面深化改革的第一年。从今年的发展环境看，世界经济持续复苏的基础和动力不稳固，我国经济增速放缓，中西部地区与我市的同质化竞争日益加剧。特别是我市正处于爬坡过坎、攻坚转型的关键时期，既面临着赶超发展、缩小差距的繁重任务，又面临着调结构、促转型和保护环境、改善民生的巨大压力。但也要看到，我国经济长期向好的基本面没有改变，全面深化改革将释放出新的动力和活力；随着全省三大战略的深入实施和我市“三化”进程的加快推进，基础支撑条件更加完备，要素保障能力不断增强，综合竞争优势日益彰显，有利于我们承接产业转移、发展新兴业态、加快发展步伐。因此，我们既要坚定信心，抢抓机遇，充分用好有利条件；又要沉着冷静，保持清醒，积极应对复杂局面，开拓进取、扎实工作，努力夺取富民强市、加快崛起的新胜利!

今年政府工作的总体要求是：全面贯彻落实党的十八大和十八届二中、三中全会精神及市委决策部署，把改革创新、扩大开放贯穿于经济社会发展各个领域各个环节，“围绕一个目标、完善两大机制、突出三项重点、落实四项要求、强化五个保障”，牢牢把握科学发展这个第一要务，全面实施开放招商这一战略，紧紧抓住投资拉动这个关键，稳增长、调结构、促改革、惠民生，促进经济社会持续健康发展，努力打造富强驻马店、文明驻马店、平安驻马店、美丽驻马店。

今年经济社会发展的主要预期目标是：生产总值增长9.5%左右，地方公共财政预算收入增长13%，规模以上工业增加值增长15%，固定资产投资增长21%，社会消费品零售总额增长14%，城乡居民收入增长9%，城镇化率提高2个百分点左右，城镇新增就业6万人，人口自然增长率控制在6.5‰以内，居民消费价格涨幅控制在3.5%左右，节能减排完成省定目标。重点抓好以下十个方面的工作：

(一)强力实施开放招商，实现招商引资新突破。坚持把开放招商作为“一举求多效”的综合性举措，大力实施开放带动战略，力争全年引进市外资金400亿元以上，其中引进省外资金215亿元以上，实际利用外资和对外贸易分别增长10%以上。突出产业招商。依托我市优势产业和现有企业，重点围绕产业上下游延链补链招商引资，拉长产业链条，做大做强食品、能源化工、装备制造、生物医药、建材、轻纺六大产业集群。引导县区发挥自身优势，着力引进一批产业关联度大、成长性好的项目集聚发展，逐步培育形成各具特色的产业集群。着力招大引强。以承办中国“农洽会”为载体，瞄准中外500强及行业领军企业，继续开展大招商活动，集中力量引进一批旗舰型企业和50亿元以上的基地型、龙头型大项目。继续推进与华润集团、中国通用、昊华集团、泰国正大、平煤集团等大企业的战略合作，进一步深化合作领域，促使企业转型升级、做大做强。拓宽招商领域。按照非禁即入的原则，对国家法律法规未明确禁止的领域一律对外开放，重点围绕城镇基础设施、现代农业、教育卫生、文化旅游、健康养老等领域，有针对性地包装一批重大项目，搞好对外发布和推介，吸引社会资本开发建设，形成全方位、宽领域的开放招商新格局。创新招商方式。更加注重驻地招商和专业招商，组建专业队伍，设立招商窗口，常年开展有针对性的招商活动。特别是对一些重大项目，主要领导亲自挂帅，组成专业团队进行定点招商，力争取得更多成果。提高招商实效。继续实行县区主要领导“二分之一”工作法，进一步完善招商引资工作考评体系，坚持实事求是，严禁弄虚作假、以少报多、以小报大和以招商为名圈占土地行为。进一步强化招商责任，搞好跟踪问效，着力提高项目履约率和资金到位率，推动招商引资工作再上新台阶。

(二)大力实施项目带动，不断增强发展后劲。深入开展项目建设推进年活动，努力扩大投资规模，优化投资结构，增强发展后劲。今年初步安排市重点建设项目275个，总投资2131亿元，年度计划投资614亿元。通过实施项目建设，实现全市重点项目投资突破500亿元、产业集聚区固定资产投资突破800亿元。加快推进重点项目建设。对天方药业和华中正大搬迁改造等计划开工项目，加大协调力度，落实开工条件，确保尽快开工建设；对昊华骏化合成氨原料路线改造、恒兴数控产业园、上蔡闽商工业园、汝南绿佳新能源、西平光电产业园、平舆祥鸿制革产业园等一批在建项目，加快建设进度，确保完成投资计划；对海川电子玻璃、中多铝业、富兴电子、联顺食品、恒丰车轮等一批计划竣工项目，倒排工期，加快进度，确保尽快竣工投产。切实加强项目谋划工作。认真研究国家出台的一系列政策，围绕节能环保、城市基础设施、现代农业、健康服务、养老产业等重点领域和产业，谋划包装一批项目对外发布，实施定向招商。引导市属重点企业围绕延链补链、转型升级，认真谋划一批重大项目，使企业备足后劲、持续发展。认真抓好项目落地。对去年中国“农洽会”和专业招商活动签约的项目，进一步明确责任，搞好跟踪问效，着力提高项目履约率和资金到位率。对宿鸭湖生态旅游开发、德威电缆产业园等重大项目，进一步洽谈对接，促使项目尽早落地。对驻马店正大百万头生猪全产业链、平煤蓝天原料替代改造、吴桂桥煤矿煤气化和洗煤厂等项目，加快前期工作，争取早日签订合作协议。进一步完善项目推进机制。继续落实重大项目联审联批、领导分包和周例会制度，进一步完善项目建设考核办法，加强项目督导检查，切实优化建设环境，确保重点项目顺利实施。

(三)科学推进新型城镇化，不断提高城镇发展承载力。认真贯彻中央和省委各项决策部署，按照我市科学推进城镇化的实施意见，坚持“以人为本、优化布局、生态文明、传承文化”的原则，积极稳妥推进新型城镇化发展。强化规划引导控制。充分发挥规划的龙头作用，认真执行新一轮城市总体规划，按照山水园林、生态宜居的定位，突出地方特色，体现绿色低碳，全面完成中心城区19个专项规划编制和报批工作；根据城市新的功能分区，加快新一轮控制性详细规划的编制。完成县乡总体规划和相关专项规划编制工作。严格执行规划，充分发挥各级规划委员会的作用，项目按规划审批，城市按规划建设，维护规划的权威性和严肃性。深入开展“双违”整治活动，坚决杜绝违法占地、违法建设行为。加强中心城区建设与管理。以推进七大功能片区、十项重点工程、六个区域综合改造和六项惠民实事为重点，全面加快中心城区建设步伐。继续推进七大功能片区建设，基本完成高铁站东广场一级开发任务；全面推进职教园区、北部新区、小清河商务休闲区和练江河养生休闲商住区基础设施建设，启动高铁西广场区建设。继续抓好十项重点工程建设，完成中原大道北段、淮河大道东段、文明大道南段及创业大道、盘龙山路等项目，加快纬十路、市体育中心和第二水源地建设。加大中心城区路网、地下管网、绿化、环卫等基础设施建设力度，抓好铜山大道、汝河大道延伸和解放大道、乐山大道改造等在建项目，完成已建成道路绿化和五座过街天桥建设。第三污水处理厂、中水厂建成投用；开工建设香山生活垃圾处理场和练江河治理二期工程，启动第四污水处理厂项目前期工作，力争中心城区基础设施建设投资在70亿元以上。实施好六个区域综合改造项目，加快红星美凯龙城市综合体建设进度，积极推进联碧德文化综合体、中心商务区南片区、朱氏河、城区南出口和东出口等区域连片开发，完成中心城区36个棚户区改造项目；加快群众安置房建设，开工建设群众安置房2.1万套、258万平方米。从完善城市功能、解决市民最关心的问题做起，继续实施好中心城区六项惠民实事，完成5个道路路口升级改造、6条道路路灯照明和2个游园建设，新建垃圾中转站5座、公厕17座，改造背街小巷道路5条，新建菜市场5个，新建和扩建中小学5所，建成市殡仪服务中心，开工建设市殡仪馆。完善城市管理长效机制，积极推进智慧城管项目建设，发挥三个区的主体作用，划小网格、重心下移，加强检查、严格考评，不断提高城市管理水平和市民的满意度。推进城乡一体化示范区建设。编制完成示范区总体规划、各项专项规划和控制性详细规划，全面开工商务中心区建设，抓紧启动都市生态农业示范区、休闲度假旅游功能区建设，大力发展金融、高端商贸、农业会展博览、物流以及生态旅游、健康养生等产业。尽快打通雪松大道、开源大道、商桐路三条骨干道路，形成示范区发展框架。加快一中心五组团发展。积极推进中心城区与遂平、汝南、确山县城以及宿鸭湖、西部山区两个生态功能区组团发展，大力实施组团连通融合工程，实现中心城区与组团之间快速通联，构筑组团式、生态型、现代化城市空间格局。推进铜山大道、天中山大道、驿城大道、文明大道等快速通道改造提升，打造中心城市组团半小时交通圈和全市1小时交通圈。加快县城和重点镇建设。全面实施县城扩容提质工程，加快基础设施建设，拉大县城框架，强化产业带动、住房和教育牵动，推动产城互动发展。选择人口规模较大、发展基础较好的县，有序推进县改市步伐。按照完善功能、体现特色的要求，重点抓好10个区位条件好、产业基础强、发展潜力大的示范镇建设。同时每个县区选择2~3个中心镇，因地制宜，分类指导，加快开发建设步伐，使之成为集聚产业和人口的重要载体。有序推进农村人口转移。深化户籍制度改革，放宽城镇入户条件，在全市实行居住证制度。切实保障进城农民土地承包经营权、农户宅基地用益物权和农民集体经济收益分配权，消除农民进城后顾之忧。加强城镇学校、医院等公共服务设施建设，稳步推进城镇基本公共服务常住人口全覆盖，切实解决好转移人员的安居、子女就学、社会保障等问题，使之进得来、落得住、过得好。积极稳妥推进新农村建设。按照“产业为基、就业为本、生计为先”原则和“五规合一”要求，尊重群众意愿，实施分类指导，自下而上、以县为主，科学编制新农村建设规划。在抓好原有试点的基础上，按照规划、量力而行，在条件成熟的地方再开工一批新农村建设项目。同时，加强基础设施和配套建设，全面改善农村生产生活条件。

(四)加快推进新型工业化，做大做强工业经济。坚持把工业发展作为首要任务，认真实施“保速增效、转型升级”行动计划，大力推进“两化”融合，努力提高产业质量，不断强化工业化主导作用。加快骨干企业和优势产业发展。筛选30家重点企业，积极推进创新驱动，深入开展战略合作，大力实施品牌带动，着力培育一批规模大、品牌影响力广、竞争力强的骨干企业，力争实现主营业务收入超100亿元企业1家、超50亿元企业4家、超10亿元企业25家。依托重点骨干企业，引进上下游和关联配套企业，着力培育大型产业集群，力争食品、轻纺、能源化工、装备制造、建材、医药六大行业主营业务收入分别在550亿元、500亿元、380亿元、380亿元、350亿元、80亿元以上。大力培育高成长性企业和战略性新兴产业。确定30家高成长性企业，积极争取上级技改项目资金支持，实施30个以上“强、高、快”重大转型升级项目，以增量投入带动存量优化，促进行业产品由产业链低端向高端提升，形成全产业链竞争优势。确定30家战略性新兴产业重点企业，加大培育和扶持力度，加快华润风电、银泰电动汽车、海川电子玻璃、富兴电子等重点项目建设，推动信息技术、新能源、新材料、节能环保、高端装备制造、生物医药等战略性新兴产业发展，力争实现增幅35%以上。加快推进产业集聚区建设。继续抓好道路、供水、供电、供气、通信等基础设施和“一中心、三平台”建设，加快建设一批公租房、学校、文化娱乐等配套设施，进一步完善功能，提高承载能力。坚持“四集一转”，严格投资强度和准入门槛，不断提高土地利用效率，原则上不在产业集聚区外摆放工业项目。深入开展产业集聚区晋档升级活动，加快培育电子玻璃、电子信息、电动汽车、新能源电动车、皮革皮具特色产业园区发展，力争市产业集聚区主营业务收入在350亿元以上，装备产业集聚区和平舆、泌阳产业集聚区在200亿元以上，上蔡、确山、遂平产业集聚区在150亿元以上，汝南、西平、正阳、开发区产业集聚区在100亿元以上。强化企业服务。推动企业服务常态化、制度化、长效化，切实帮助企业解决实际困难。进一步完善要素保障机制，引导资金、土地、人才等要素资源向重点企业、重点项目集中，破解企业发展瓶颈。切实加强经济运行监测和协调，搞好煤、电、油、气、运协调保障，确保工业经济平稳运行。

(五)积极推进农业现代化，做强做优现代农业。按照“抓基础、增后劲，抓产业、增效益，抓改革、增活力”的总要求，全面落实强农惠农政策，实施好重大项目和关键举措，努力实现农业大市向农业强市、传统农业向现代农业转变。大力实施高标准粮田“百千万”工程。作为粮食主产区，确保国家粮食安全是我们的政治责任。抓住国家构建粮食安全战略的机遇，加快高标准粮田建设和中低产田改造，持续推进千亿斤粮食、农业综合开发、小农水重点县、土地整理等工程建设，重点实施好9个集中连片5万~10万亩高标准粮田项目区，力争新增高标准粮田120万亩。大力实施现代农业产业化集群培育工程。重点围绕粮食、油料、生猪、肉牛、家禽、林果等农产品资源，加强标准化规模种植养殖基地建设，培育壮大龙头企业，逐步打造“全链条、全循环、高质量、高效益”的现代农业产业集群，力争省级农业产业化集群达到15个、市级达到30个。启动实施都市生态农业发展工程。重点在中心城区和确山、汝南、遂平3个组团县城周边，以特色种植业、设施园艺业、生态休闲业为主体，规划建设一批集特色高效农产品生产基地、休闲观光旅游和“菜篮子”工程为一体的都市生态农业工程，今年重点做好规划工作，并启动实施一批重点项目，确保尽快见到实效。积极培育新型农业经营体系。加快土地流转服务平台建设，鼓励支持承包土地向专业大户、家庭农场、农民合作社流转，推进多种形式规模经营，力争全市土地流转面积占比在20%以上。鼓励引导社会资本和其他生产要素有序进入农业和农村，重点培育9个农民合作社示范乡镇、新增1000家农民合作社和1000家家庭农场、培育创建100家市级示范社、组建100家联合社。实施精准扶贫开发。建立健全精准扶贫工作机制，重点抓好产业扶贫、专项扶贫、行业扶贫、社会扶贫，促进贫困乡村产业发展，减少贫困人口数量，增加贫困人口收入，改善生产生活条件，力争完成14.5万人的脱贫任务。

(六)加快发展服务业，做大做活第三产业。把加快发展服务业作为产业转型升级的重点，既重视生活性服务业，又重视生产性服务业；既抓好传统服务业，又抓好现代服务业，促进服务业发展提速、比重提高、水平提升。加快商务中心区和特色商业区建设。全面启动市商务中心区核心区高档楼宇建设，加快推进红星美凯龙、建业十八城等重大项目，努力打造以高铁站前广场为核心、辐射周边的大型商务中心圈。加快平舆、上蔡、泌阳、正阳商务中心区和驿城区、遂平、西平、汝南、确山特色商业区建设，力争全市“两区”建设投资在90亿元以上。促进房地产业健康发展。落实差别化信贷和税收政策，加强土地计划调控，创造良好环境，着力引进一批知名房地产企业连片开发建设，促进房地产投资持续增长。不断优化住房供应结构，增加中低价位、中小套型普通住房供应，满足多数群众的住房需求。加快文化旅游产业发展。积极推进大嵖岈旅游区、西部山区和宿鸭湖生态旅游组团、乡村旅游产业带开发建设，大力开拓旅游市场，提升服务质量，力争全市A级旅游景区超过20个，实现5A级旅游景区零的突破，旅游业综合收入突破100亿元。加快文化产业园区和示范基地建设，推动演艺、网络、工艺美术等文化产业发展。加快现代物流业发展。完成物流业发展规划，申报筹建“郑州海关驻马店办事处”，力争公共保税中心上半年封关运行；加快实施火车站货场北迁和大刘庄物流园区建设，推进粮食、化工、冷链、快递等产业物流发展，支持企业建立电子商务平台，规划建设市电子商务物流园区。继续实施“万村千乡”工程，积极开展农超对接、农社对接，推动物流网点、物流配送向农村延伸，着力打造豫南区域物流中心。加快健康养老产业发展。编制完成养老服务业十年发展规划，规划建设健康养老产业园，加快实施康复护理院等项目建设，全面推进城乡社区居家养老服务和农村幸福院建设，力争全市新建养老服务机构19所，完成投资16亿元。进一步完善家庭服务业发展配套措施，着力打造一批有影响力的知名家庭服务企业。

(七)持续推进生态文明建设，着力打造美丽驻马店。正确处理发展经济和保护环境的关系，把生态文明建设放在更加突出位置，强力推进生态文明建设。强化生态资源保护。认真落实属地管理责任，不断完善生态补偿机制，实行最严格的源头保护制度、损害赔偿制度、责任追究制度，切实增强可持续发展能力。加强林业生态建设，抓好天然林保护、退耕还林、水土保持、市城郊防护林等项目建设，确保完成造林20.8万亩，森林抚育和改造12.5万亩。巩固西部山区“三乱两超”综合整治成果，严格按划定区域进行分类管理，坚决打击私采乱挖行为。加强生态环境治理。实施“蓝天工程”，落实好国家大气污染防治措施，推动重点行业脱硫脱硝除尘改造，强制推进集中供热覆盖区域小锅炉关停，加强机动车尾气治理、建筑施工和道路扬尘防治、秸秆焚烧整治，有效遏制大气污染。实施“碧水工程”，抓好市产业集聚区和西平、汝南、泌阳、遂平、正阳、确山等县第二污水处理厂建设，强化企业污染排放监管，严格落实水资源管理制度，加强饮用水源地保护；扎实开展城市河流清洁行动，积极推进练江河、黑河等河流综合整治，加快实施宿鸭湖水库生态修复工程，切实改善水环境质量。实施“乡村清洁工程”，积极推进农村环境综合整治，加强农村面源污染治理，开展美丽乡村创建，改善农村人居环境。推进生态城市创建。制定生态市建设规划，建立健全财政投入和社会资本融资相结合的投入机制、科学合理的奖惩和干部考核机制以及主要领导牵头、各方齐抓共管的领导机制，全面启动中心城区环保模范城和生态市创建工作。搞好节能减排。全面推进重点领域节能降耗，加快淘汰落后产能，严格控制高耗能、高污染项目上马；强力推动重点减排工程建设和原有减排项目监管，严格落实污染物排放总量预算管理办法，确保完成省定目标任务。通过综合施策、常抓不懈，保护好我们的碧水蓝天，人民群众呼吸上新鲜空气、喝上放心水，驻马店天更蓝、水更清、地更绿，山川更秀美、城乡更宜居、更加美丽可爱。

(八)完善基础支撑条件，加快培育发展新优势。加强支撑体系建设，提升发展层次、拓展发展空间，增强长远发展后劲。强化基础设施支撑。加快京港澳高速驻马店段改扩建进度，开工建设京港澳高速驻马店北出口、开源大道出口改造和驻马店高铁客运枢纽站，积极实施107国道升级改造和正阳、泌阳、上蔡等通行条件较差地区干线公路改造；大力发展城市公交，加快中心城区公交首末站、换乘站建设，缓解城市交通拥堵压力；抓住机遇，积极实施农村公路“乡村畅通”工程三年行动计划，打好农村公路建设翻身仗，力争全年改造建设农村公路600公里，完成交通项目总投资25亿元。加快病险水库除险加固、大型灌区续建配套及节水改造等水利工程建设，新增和恢复有效灌溉面积60万亩、旱保田50万亩，新增和改善除涝面积60万亩。加快驻北500千伏输变电工程建设，扎实推进农村电网改造升级。积极推进“宽带驻马店”、“智慧驻马店”等信息化项目建设，促进信息化与“三化”融合发展。强化要素保障支撑。大力实施全民技能振兴和职业教育攻坚工程，积极开展订单培训，完成各类职业技能培训18万人次，新增农村劳动力就地就业1万人。加强银企对接，扩大信贷投放和融资规模，全年新增贷款120亿元以上；做好上市后备企业培育工作，重视融资平台建设，提高直接融资能力。完善土地保障机制，加强土地计划调控，优先支持重点项目和民生项目用地，大力实施农村土地综合整治，积极拓展人地挂钩成果，推进土地节约集约利用。强化科技创新支撑。认真落实科技创新政策，实施企业创新能力培育工程，突出企业创新主体地位，积极培育高新技术企业，实施重大科技专项，突破一批关键核心技术，力争新创建一批国家、省级企业技术中心、工程技术研究中心。强化发展环境支撑。深入开展优化环境专项治理活动，严厉打击破坏发展环境的行为，特别是对那些执法犯法、吃拿卡要的典型案件，以及封门堵路、敲诈勒索、干扰施工、恶意阻扰项目建设的组织者、操纵者和黑恶势力，要硬起手腕，坚决依法打击!

(九)全面深化改革，不断增强发展内生动力。认真贯彻十八届三中全会精神，坚定不移地深化重点领域和关键环节的改革。深化国有企业改革。力争今年基本完成全市国有企业改制任务。积极发展混合所有制经济，进一步推进股份制改革，深化企业内部三项制度改革，完善法人治理结构。积极探索国有资本授权经营体制改革，开展组建或改组国有资本运营、投资公司试点。深化财税体制改革。完善全面规范、公开透明的财政预算制度，加强预算绩效管理，强化增收节支，严格控制“三公经费”支出。清理规范税收优惠政策，全面落实“营改增”、资源税从价计征等改革政策。深化金融体制改革。加快地方金融机构改革发展，积极引进股份制银行入驻，推动符合条件的县域农信社组建为农商行，加快村镇银行组建步伐。探索土地股份和银行授信等新型农村金融融资模式。积极配合中原银行组建工作。推动保险业拓宽服务领域，提高服务质量。加强排查监管，完善工作机制，严厉打击非法集资活动，防范和化解金融风险。深化医药卫生体制改革。统筹推进医疗保障、医疗服务、公共卫生、药品供应、监管体制改革，巩固基层医疗卫生机构综合改革成果，完善城乡基层医疗卫生服务运行新机制，抓好公立医院改革，鼓励社会办医。全面深化农村改革。坚持完善农村基本经营制度，不断探索农村土地集体所有制有效实现形式，探索建立兼顾国家、集体、个人的土地增值收益分配和宅基地、责任田有偿退出机制，继续深入推进集体林权制度改革，加快推进农村土地承包经营权确权颁证，年底前完成农村宅基地确权登记颁证工作。

(十)切实保障和改善民生，促进社会和谐稳定。把保障和改善民生作为政府工作的根本出发点和落脚点，继续加大投入力度，实施好十项重点民生工程，使发展成果更多地惠及人民群众。大力发展各项社会事业。启动二期学前教育三年行动计划，稳妥推进农村中小学布局调整，加快城区中小学建设，着力解决“入学难”、“大班额”问题，推动义务教育均衡发展。支持特殊教育发展。加快市职教园区建设，力争完成投资15亿元以上，完成幼儿师范、驻马店农校和卫校一期工程建设并搬迁入住；深化校企合作，着力打造一批优质特色职业院校；全力办好教育部中国特色应用技术大学“驻马店论坛”；落实优惠政策，积极发展民办教育。积极推进医疗卫生事业三年九大攻坚行动计划，加快中心医院北区分院、妇女儿童医院建设进度，抓好国家重大疾病控制、县级医院和乡镇卫生院改扩建等项目，着力提高城乡基层卫生服务标准化建设水平。加强残疾人就业培训工作，落实好0~6岁残疾儿童抢救性康复任务，确保残疾人康复中心建成投用。继续实施文化惠民工程，切实加强基层文化基础设施建设，加快报业大厦项目建设进度，促进文化产业加快发展。大力发展全民健身事业，加快体育健身设施建设，积极参加十二届省运动会和首届全民健身大会。认真落实生育政策，稳定低生育水平，促进人口长期均衡发展。加强就业和社会保障工作。完善就业创业公共服务体系，加强基层人力资源服务平台建设，鼓励支持民营经济和中小微企业发展，发挥创业带动就业的倍增效应，确保全年城镇新增就业6万人，下岗失业人员再就业1.8万人，新增农村劳动力转移就业7万人。全面实现失业保险市级统筹，完善医疗保险市级统筹，继续推进全市就医一卡通，抓好城乡居民大病保险试点。巩固城乡居民社会养老保险成果，扩大社会保险覆盖范围，确保各项社会保险待遇按时足额发放。继续提高城乡低保、农村五保供养和优抚对象待遇，不断完善城乡社会救助体系，兜住保障困难群众基本生活底线。继续扩大住房公积金征缴范围，适当提高缴存比例。加大保障性住房建设力度，全年建设保障性住房项目61个3.68万套。大力发展社会福利和慈善、老龄、妇女儿童、红十字等事业。加强和创新社会治理。深入推进平安驻马店建设，做好社会矛盾排查化解工作，进一步强化和完善社会稳定风险评估机制，着力解决群众反映强烈的信访问题。做好第八届村委会换届选举工作，加强社会诚信体系建设。加强法制宣传教育，做好法律援助工作。完善和创新流动人口、特殊人群的管理服务。严格落实安全生产责任，深化重点行业和重点领域整治，以更坚决、更有力的措施，消除安全生产隐患，坚决遏制重特大事故发生。加强社会治安综合治理，依法严厉打击违法犯罪活动。全面推进食品安全监管体制改革，建立健全食品药品、农产品、畜产品安全保障体系和监管体系，强化安全监管责任和责任追究，用严格的标准、严密的监管、严厉的惩处、严肃的问责，确保人民群众“舌尖上的安全”。

继续加强国防教育和国防后备力量建设，支持驻驻解放军、武警部队、预备役部队、民兵和人民防空建设，深入开展双拥共建，巩固军政军民团结，促进军民融合式发展。支持工会、共青团、妇联等人民团体发挥桥梁纽带作用，注重发挥工商联等社团作用。加快气象现代化建设，搞好气象服务。继续做好人事、审计、统计、民族宗教、外事侨务、档案、史志、地震等工作。

三、切实加强政府自身建设

把转变政府职能、强化依法行政、提升能力素质作为政府自身建设的关键，深入开展两转两提，努力建设法治政府、服务政府、高效政府、创新政府、廉洁政府，不断提升政府工作水平。

(一)加快转变政府职能。围绕市场在资源配置中起决定性作用和更好发挥政府作用，推动政府职能向创造良好发展环境、提供优质公共服务、维护社会公平正义转变。进一步简政放权，持续清理行政审批事项，把该放的权力坚决放到位，把该管的事情坚决管住管好，对保留的审批事项简化手续、优化流程、在线运行、限时办结。推进工商注册制度便利化，由先证后照改为先照后证。扎实推进政府机构和事业单位分类改革，基本完成承担行政职能事业单位改革，推行政府购买社会服务。严格机构编制管理，确保编制和财政供养人员只减不增。

(二)深入推进依法行政。严格按照法定权限和程序行使权力、履行职责，自觉用法治思维和方式调节经济运行、管理社会事务。坚持规范、公正、文明执法，充分发挥行政监察和审计监督的作用，认真落实执法责任制和纠错制度。完善依法行政各项制度，建立和完善群众参与、专家咨询和集体决策相结合的决策机制，坚持用制度管权、管事、管人，不断提高政府公信力和执行力。做好行政调解等工作，办好行政复议案件。深入开展法制宣传教育和公务员培训，建设高素质的公务员队伍，不断提升服务质量和公务员的依法行政能力。

(三)大力改进工作作风。坚持人民至上，始终把人民群众放在心中最高位置，始终把群众满意作为第一工作标准，认真落实中央八项规定和省市实施意见，扎实开展党的群众路线教育实践活动，从市政府领导班子做起，以上率下，坚决纠正“四风”。坚持一切从实际出发，脚踏实地，务实重干，不急功近利，不盲目跟风，努力做出经得起实践、人民、历史检验的业绩。坚持令行禁止，加强行政督查，着力提高办事效率和行政效能。坚持简约从政，严格执行领导干部办公用房、公务用车等有关规定，杜绝铺张浪费，压缩一切不必要开支，集中财力办大事，确保把有限的财力用到最急需的地方。认真落实党风廉政建设责任制，完善惩防体系，加强制约监督，规范权力运行，坚决惩处权钱交易等违法违规行为，努力做到干部清正、政府清廉、政治清明。

各位代表!作为人民政府，我们一定严格执行市人大及其常委会的决议决定，自觉接受法律和工作监督，主动接受市政协的民主监督，虚心接受人民群众社会监督和媒体舆论监督，认真办理人大代表建议和政协提案，广泛听取各民主党派、工商联、无党派人士、人民团体的意见，让人民更有效地监督政府，使政府更好地服务人民，决不允许慵懒散奢，决不允许弄权渎职，决不允许损害群众利益!

各位代表!面对改革发展的新形势，面对全市人民的新期待，我们深感责任重大、使命光荣。让我们在省委、省政府和市委的正确领导下，进一步解放思想、开拓创新、扎实工作，凝聚全市人民的智慧和力量，努力打造富强驻马店、文明驻马店、平安驻马店、美丽驻马店，不断开创富民强市加快崛起的新局面!
